# Advancements in Circulating Tumor Cell Detection for Early Cancer Diagnosis: An Integration of Machine Learning Algorithms with Microfluidic Technologies

**DOI:** 10.3390/bios15040220

**Published:** 2025-03-29

**Authors:** Ling An, Yi Liu, Yaling Liu

**Affiliations:** 1 School of Engineering, Dali University, Dali 671003, China; anling@stu.dali.edu.cn; 2 Precision Medicine Translational Research Center, West China Hospital, Sichuan University, Chengdu 610041, China; 3 Department of Bioengineering, Lehigh University, Bethlehem, PA 18015, USA

**Keywords:** circulating tumor cells (CTCs), microfluidic systems, machine learning algorithms, early cancer diagnosis

## Abstract

Circulating tumor cells (CTCs) are vital indicators of metastasis and provide a non-invasive method for early cancer diagnosis, prognosis, and therapeutic monitoring. However, their low prevalence and heterogeneity in the bloodstream pose significant challenges for detection. Microfluidic systems, or “lab-on-a-chip” devices, have emerged as a revolutionary tool in liquid biopsy, enabling efficient isolation and analysis of CTCs. These systems offer advantages such as reduced sample volume, enhanced sensitivity, and the ability to integrate multiple processes into a single platform. Several microfluidic techniques, including size-based filtration, dielectrophoresis, and immunoaffinity capture, have been developed to enhance CTC detection. The integration of machine learning (ML) with microfluidic systems has further improved the specificity and accuracy of CTC detection, significantly advancing the speed and efficiency of early cancer diagnosis. ML models have enabled more precise analysis of CTCs by automating detection processes and enhancing the ability to identify rare and heterogeneous cell populations. These advancements have already demonstrated their potential in improving diagnostic accuracy and enabling more personalized treatment approaches. In this review, we highlight the latest progress in the integration of microfluidic technologies and ML algorithms, emphasizing how their combination has changed early cancer diagnosis and contributed to significant advancements in this field.

## 1. Introduction

**Figure 1:**
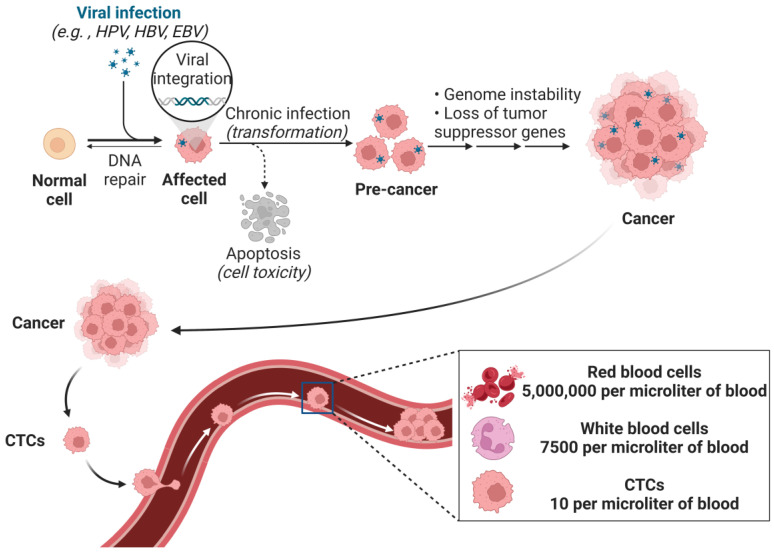
Cancer cell metastasis process and the relative amount of CTCs in the blood. Viral infection causes normal cells to transform into cancer cells, which break off from the tumor and enter the bloodstream, forming CTCs. (Created with BioRender.com).

Cancer remains a leading cause of death worldwide [1]. The global cancer burden is expected to rise to 29 million new cases annually by 2040, a 62% increase from 2018 [2]. Early and precise cancer detection remains a challenge, particularly in metastatic disease, which accounts for over 90% of cancer-related deaths [3]. CTCs, shed from primary tumors into the bloodstream, play a key role in metastasis. CTCs offer critical insights into tumor progression and serve as biomarkers in liquid biopsies for detecting cancers like breast, prostate, and liver cancer [4,5]. However, as shown in Figure 1, the low concentration of CTCs in blood makes their detection challenging, as only a few CTCs are found among billions of blood cells [6,7,8]. In 1869, Professor Ashworth first identified CTCs in the bloodstream of a cancer patient [9]. Despite the passage of over 150 years since the initial discovery and characterization of CTCs in human blood, research on CTCs remained sparse until the mid-1990s [4]. In recent decades, several methods have been developed for isolating and detecting CTCs based on their unique physical and biological properties [10]. Traditional methods include density-based separation (e.g., FicollHypaque), size-based separation (e.g., ISET), flow cytometry, and immunomagnetic separation (e.g., CellSearch). Currently, CellSearch is the only FDA-approved system for CTC detection in breast, colorectal, and prostate cancers [11]. However, traditional techniques face challenges such as low sensitivity, potential cellular damage, and high cost [12,13]. Recently, microfluidic technology and ML have emerged as innovative solutions to improve CTC detection’s sensitivity and specificity [14].

Microfluidic technology is a burgeoning field with many advantages. These include compactness, minimal sample and reagent requirements, short analysis duration, enhanced efficiency, superior sensitivity, precise manipulation, miniaturization, and portability [15]. The geometry of microfluidic channels suits cell dimensions, enabling operations like modulating the cellular environment and conducting single-cell analyses [16]. Over the past decade, microfluidic platforms with functional microchannels have been developed for CTC isolation [17]. These platforms streamline sample collection, loading, separation, and analysis, reducing processing time and improving CTC capture. They can modulate cell–antibody interactions by controlling fluid flow and enhancing capture efficacy, and they can incorporate materials like ceramics, metals, and polymers to improve detection efficiency.

ML, a branch of artificial intelligence, has transformed data interpretation in biomedical research. The FDA has endorsed several commercial products that incorporate ML algorithms for medical diagnosis and research [18]. ML is increasingly used with microfluidic devices to decode complex data [19]. While CTC enrichment and screening can be automated, enumeration still requires manual intervention, risking human error [20]. ML-powered medical image analysis can improve automation. It also plays a key role in developing automated classification and detection methods. Conventional models, like random forests, classify based on features. Deep learning models, like convolutional neural networks (CNNs), learn features autonomously [21]. For example, CNNs have been deployed in classifying cell lines and red blood cells [22,23]. The synergy between ML and microfluidic technology signifies not merely an incremental advancement but heralds a paradigmatic transformation of the methodologies for CTC detection [24].

The aim of this review is to examine the roles of microfluidic technology, ML, and their integration in early cancer detection. The combination of these technologies has the potential to improve the specificity and sensitivity of CTC detection, as well as enable real-time monitoring and predictive analytics [25]. However, challenges remain, such as the complexities of algorithm training and microfluidic design [26]. Despite these challenges, the integration of microfluidic technology and ML may offer promising advancements in CTC detection and precision oncology, contributing to the development of more effective tools for early cancer detection.

## 2. Microfluidic Technology and Its Significance in CTCs

### 2.1. Fundamentals of Microfluidic Technology

Microfluidic technology, which enables precise manipulation of fluids at the microscale (typically within 10–500 μm channel dimensions), has emerged as a cornerstone of lab-on-a-chip systems by integrating fluid dynamics with microfabrication techniques [27,28]. Originating from semiconductor miniaturization, this field utilizes photolithography and soft lithography to create three-dimensional microchannels with unique hydrodynamic properties distinct from macroscopic systems [29,30]. At the microscale, viscous forces dominate over inertial forces, resulting in laminar flow patterns. This leads to predictable fluid behavior, minimal mixing, and precise control of parameters such as flow rate and pressure [31,32]. Recent innovations have focused on material science and computational optimization. Polydimethylsiloxane (PDMS)-based materials remain widely adopted for their biocompatibility and ease of fabrication, while advancements in stimuli-responsive hydrogels now enable dynamic flow control through thermomechanical actuation without external devices [33,34,35,36]. Concurrently, ML has become a powerful tool in optimizing microchannel design. Neural networks trained on experimental flow data can predict optimal geometries for complex fluid operations, significantly reducing prototyping time compared to traditional methods [37,38].

**Figure 2:**
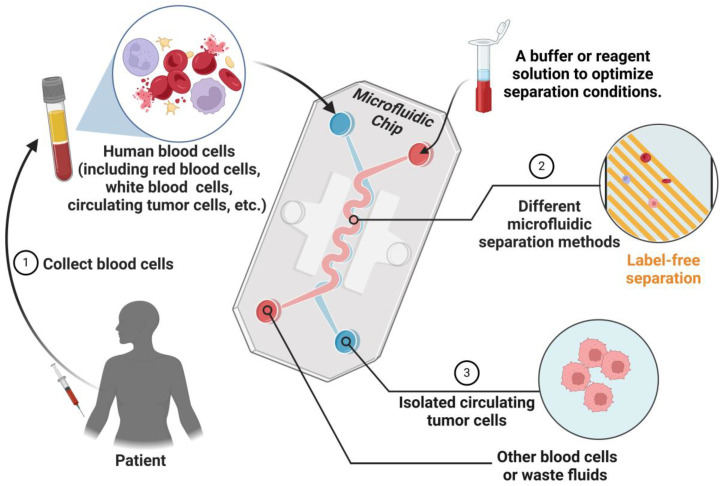
Schematic diagram of microfluidic system separating CTCs. Blood samples containing red blood cells, white blood cells, and CTCs collected from patients are sent to the microfluidic chip. The chip employs a variety of label-free microfluidic separation methods. The central serpentine main channel can be designed to integrate multiple microfluidic techniques, such as inertial separation, magnetic separation, or electric field separation, to enhance cell sorting efficiency. After the chip processing, the CTCs are successfully isolated. (Created with BioRender.com).

Microfluidic technology has emerged as a central focus within biomedical research, particularly for its pivotal role in the detection of CTCs in the realms of cancer diagnosis and treatment monitoring [39]. As shown in Figure 2, numerous microfluidic devices have been developed for the detection and isolation of CTCs, primarily employing positive selection strategies based on the epithelial marker, epithelial cell adhesion molecule (EpCAM) [40]. Among these, Micropillar CTC chips and Herringbone CTC chips are notable microfluidic platforms that utilize positive selection to isolate CTCs [25]. These systems adeptly capture CTCs on EpCAM-functionalized structures within the peripheral blood of patients with metastatic cancer, leveraging precisely controlled laminar flow phenomena. Subsequently, they facilitate the successful identification of CTCs through fluorescent immunostaining.

### 2.2. Applications of Microfluidics in CTCs Detection

CTCs represent a scant number of cancerous cells within the bloodstream, playing a crucial role in metastasis and cancer progression. Despite the significant potential of CTC detection and analysis for early cancer diagnosis, prognostication, and monitoring treatment efficacy, the extreme rarity of CTCs in blood—often just a few cells amid billions—presents substantial challenges [41]. Traditional methods of detection frequently fall short in achieving the requisite sensitivity and specificity, propelling the quest for efficacious CTC detection techniques into the research spotlight. The advent of microfluidic devices heralds a significant breakthrough in this arena. These devices offer unparalleled precision in isolating and analyzing CTCs by meticulously controlling fluid flow. Recent innovations in chip design and surface functionalization have markedly enhanced the efficiency and precision of CTC capture. Employing techniques such as immunoaffinity, size-based separation, and dielectrophoresis, microfluidic platforms have advanced the collection rate and purity of CTCs extracted from blood samples, offering promising avenues for improving cancer diagnostic and therapeutic strategies [42]. Table 1 presents the advantages, disadvantages, application scenarios, and performance metrics of microfluidic techniques for CTCs isolation and detection.

Various microfluidic platforms have been developed to enhance CTC isolation and detection through innovative capture and separation techniques. Harb et al. [43] introduced a microfluidic platform (IsoFlux) that leverages flow control and immunomagnetic capture to augment the isolation of CTCs, offering a highly sensitive approach for the capture and analysis of CTCs. Peeters et al. [44] advanced the DEPArray system, which facilitates the dielectrophoretic manipulation and isolation of single and 100%-purified CTC clusters from pre-enriched blood samples, and investigated the potential for their molecular characterization. Chen et al. [6] showcased a 3D printed CTC capture microfluidic device that significantly increases surface area by altering fluid flow patterns, thereby enhancing the contact interaction between tumor cells and 3D-printed internal structures. These internal structures are subsequently functionalized with anti-EpCAM antibodies to ensure the specific capture of EpCAM-positive human cancer cell lines, including MCF-7 breast cancer, SW480 colon cancer, and PC3 prostate cancer. The findings revealed that the CTC capture efficiencies for MCF-7, SW480, and PC3 cells were 92.42 ± 2.00%, 87.74 ± 1.22%, and 89.35 ± 1.21%, respectively. Moreover, 3D printed micro-fluidic devices have demonstrated the capability to isolate CTCs from synthetic blood samples. When integrated with enzymatic breakdown of tumor cells and subsequent analysis of liberated DNA in the blood, 3D printing microfluidic technology presents potential avenues for isolating rare tumor cells and facilitating the early detection of cancer metastasis.

**Figure 3:**
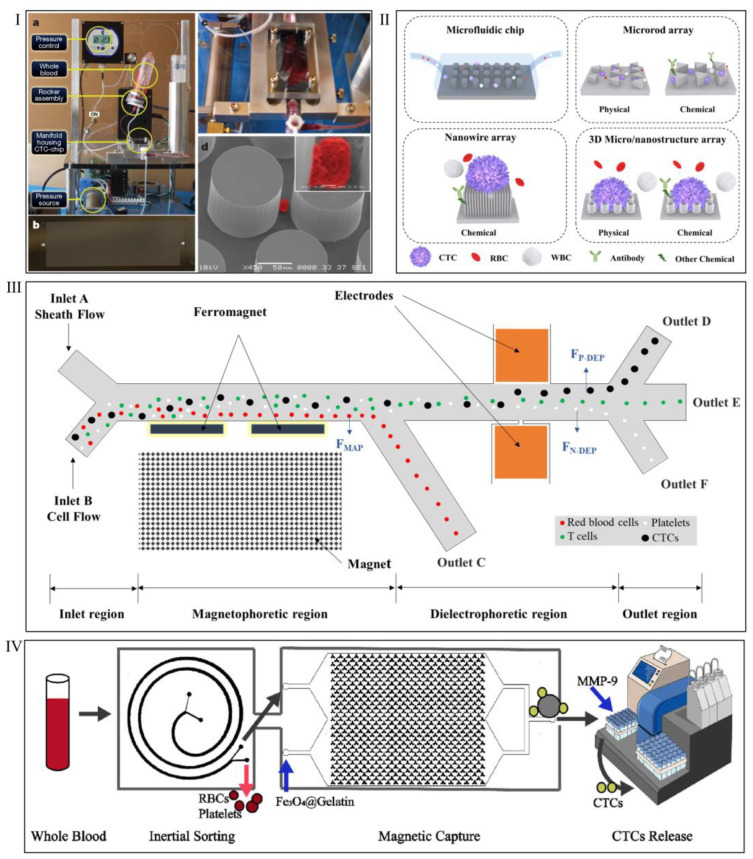
Different microfluidic devices for separating CTCs. (**I**) Isolation of CTCs from whole blood using a microfluidic device. (**a**) Schematic of the workstation configured for CTC isolation. The sample is kept in continuous motion on a rocker and delivered through the microfluidic chip via a pressure-regulated pneumatic pump. (**b**) The CTC chip, featuring an array of microposts etched into a silicon substrate. (**c**) Whole blood perfusing through the microfluidic channel. (**d**) Scanning electron microscope (SEM) image of a captured NCI-H1650 lung cancer cell spiked into blood (pseudo-colored in red). The inset shows a higher magnification of the cell. Reproduced with permission from [45]. (**II**) Schematic illustrations of micro-/nanostructure-integrated microfluidic devices for CTC capture. Reproduced with permission from [46]. (**III**) Diagram of the microfluidic channel. Reproduced with permission from [47]. (**IV**) Illustrative diagram of the microfluidic device for CTC capture and release. Reproduced with permission from [48].

Figure 3 illustrates various microfluidic devices designed for efficient CTC separation, highlighting different capture mechanisms. Nagrath et al. [45] delineated a microfluidic platform, the CTC-chip, which employs micropillar arrays on the microfluidic platform to effectively and selectively target CTCs via the interaction of antibody (EpCAM)-coated micropillars, enabling the isolation of viable CTCs from peripheral whole blood samples (Figure 3Ι). This configuration permits the efficient capture of CTCs from blood samples while allowing other cells to pass through. Figure 3ΙΙ presents schematic illustrations of micro-/nanostructure-integrated microfluidic devices designed for CTC capture, showcasing various structural configurations, including 1D microrod arrays, 1D nanowire arrays, and 3D hierarchical micro-/nanostructure arrays [46]. Zhao et al. [47] developed an integrated microfluidic chip that combines dielectrophoresis (DEP) and magnetophoresis (MAP) for the isolation of CTCs. This chip generates a strong gradient of the magnetic field through a ferromagnet structure and induces a non-uniform electric field via asymmetric orifices, enabling high-resolution separation of red blood cells, platelets, T cells, HT-29, and MDA-231 cells. Li et al. [48] developed a novel microfluidic device for the efficient capture and release of heterogeneous CTCs directly from whole blood. A spiral chip was designed to separate cells based on size, achieving a 98% recovery rate for larger CTCs. To address CTC heterogeneity, CD146-containing magnetic beads were used alongside EpCAM-based capture, increasing capture efficiency by 20%. Additionally, MMP-9 was utilized to degrade gelatin on Fe_3_O_4_ surfaces, enabling high-efficiency CTC release with minimal damage. This method successfully isolated and released CTCs from simulated patient blood, contributing to early detection and downstream clinical analysis.

One of the paramount benefits of microfluidic systems lies in their high-throughput analytical capabilities. This attribute facilitates the examination of a substantial volume of CTCs within a condensed timeframe, yielding critical insights into cancer heterogeneity, treatment efficacy, and disease progression [49]. Furthermore, the confluence of microfluidic technology with cutting-edge data analysis tools, such as ML algorithms, has ushered CTC detection into a novel epoch [50]. These synergistic systems amplify the proficiency of real-time data processing, pattern recognition, and automated decision-making, markedly enhancing the efficiency and precision of CTC detection and characterization. In conclusion, microfluidic technology has revolutionized CTC detection and analysis. Recent advancements have significantly improved sensitivity, efficiency, and throughput, making it an indispensable tool for cancer research and clinical applications. The amalgamation of microfluidics with ML presents a formidable approach for CTC detection and holds the potential to drive advancements in personalized cancer therapy [51].

## 3. ML and Its Application in CTCs

### 3.1. Fundamentals of ML

ML is a subset of artificial intelligence that enables systems to learn from data, adapt, and make predictions or decisions. It involves building algorithms that can identify patterns in data, allowing the system to generalize from prior examples and make reliable predictions for new, unseen data [52]. ML is a powerful tool for solving problems where traditional programming methods are less effective, enabling automated decision-making in a wide range of applications. Deep learning is a subset of machine learning (ML) that uses neural networks with many layers to model complex patterns in large datasets [53,54]. DL excels in tasks such as image and speech recognition, natural language processing, and even predicting cancer outcomes by analyzing complex medical data. Unlike traditional ML, which often requires manual feature extraction, DL models automatically learn to identify relevant features from raw data. This makes DL especially powerful for analyzing high-dimensional data, such as medical imaging or genomic data, where manual feature extraction is challenging. DL has rapidly advanced in fields such as computer vision, voice recognition, and cancer diagnosis, offering significant improvements in diagnostic accuracy and efficiency.

ML can be categorized into three main types based on how the model learns from data: supervised learning, unsupervised learning, and reinforcement learning. Each type of learning differs in terms of the nature of the data provided and the method used for training the model. In supervised learning [55,56], algorithms are trained using labeled data, where both the input and the corresponding output are known. The model learns to map inputs to outputs, enabling it to make predictions on new, unseen data. Unsupervised learning [57], in contrast, deals with data that do not have labels. The objective is to uncover hidden patterns or structures within the data, typically using techniques such as clustering or dimensionality reduction. Reinforcement learning is a distinct approach in which an agent interacts with an environment and learns through feedback in the form of rewards or penalties. The goal is to optimize the agent’s actions to maximize long-term rewards. Each of these learning paradigms plays a crucial role in addressing different types of problems based on the data and objectives at hand.

In ML, there are several key concepts that are crucial for understanding how algorithms function. One of the most important concepts is the distinction between training data and testing data. The training data are used to develop the model, while the testing data help evaluate its performance. Overfitting and underfitting are common challenges; overfitting occurs when a model is too complex and captures unnecessary details or noise from the training data, while underfitting happens when the model is too simple to capture the underlying patterns. Cross-validation is a technique used to improve the model’s generalization by assessing it on various subsets of the data. Furthermore, evaluation metrics such as accuracy, precision, recall, and F1-score are critical for measuring the model’s effectiveness, particularly in classification tasks.

In this review, several ML methods are discussed, each playing a vital role in the detection and analysis of CTCs. CNNs [58,59] are a type of deep learning model designed for processing structured grid-like data, such as images. They use convolutional layers to automatically extract hierarchical features from input data, making them highly effective in image classification and recognition tasks. A support vector machine (SVM) [59,60] is a supervised learning algorithm that finds the optimal hyperplane to separate different classes in a dataset, commonly used for classification problems. Random forest [61,62] is an ensemble learning method that builds multiple decision trees and combines their outputs to improve prediction accuracy and robustness. Recurrent neural networks (RNNs) [63,64] are neural networks designed for sequential data, where previous inputs influence current predictions, making them useful for tasks involving temporal dependencies. Principal component analysis (PCA) [18,65] is a statistical technique for dimensionality reduction, transforming high-dimensional data into a lower-dimensional space while retaining the most important variance. Clustering algorithms, such as K-means [66], are unsupervised learning methods that group similar data points based on shared features, helping to identify patterns in large datasets.

### 3.2. Applications of ML in CTCs Detection

In recent years, the rapid and automatic recognition of CTCs has emerged as a pivotal technology in cancer research and diagnosis [67,68]. With the advancement of scientific and technological methodologies, microfluidic platforms equipped with multifunctional microfluidic chips and unique image processing algorithms have increasingly provided new possibilities for the automatic identification of CTCs [69,70,71]. However, the high heterogeneity of CTCs introduces potential subjective limitations to these classification methods. The rapid development of artificial intelligence (AI), particularly the progress in ML techniques, offers novel solutions to overcome this challenge. ML, recognized for its objectivity, speed of processing, and robustness against noise, has been widely applied in medical research and medical image analysis. Deep learning and CNN, as classical algorithms within ML, have made significant contributions to advancing medical research, especially in cancer diagnosis and tissue identification [59].

Various ML methods, including random forests [61,62], SVM [59,60], naive Bayes classifiers [72], and k-nearest neighbors [73], have been employed in the study of CTCs. Compared to traditional ML approaches, automatic image processing methods based on CNNs can eliminate biases brought by personal subjectivity, enhancing the accuracy and objectivity of recognition. Furthermore, CNNs based on deep learning have demonstrated immense potential in CTC recognition, exhibiting sensitivities and specificities exceeding 90% [58,59]. Recent advancements in ML have significantly improved the accuracy, efficiency, and automation of CTC detection by addressing key challenges such as image-based feature classification, data imbalance, and high-dimensional complexity. Faced with the difficulty in classifying CTCs and non-cancerous cells, Zhou et al. [74] used the SVM method to classify CTCs in blood samples of breast cancer patients and predict metastasis or local recurrence based on the epithelial–mesenchymal transition (EMT) state. The model achieved a sensitivity of 77.78% and a specificity of 97.56% in the training dataset, showing its high efficiency in CTC identification. In view of the subjectivity and inefficiency of CTC microscopic image analysis, Guo et al. [25] used CNN for automatic CTC identification and trained and tested it based on immunofluorescence in situ hybridization (imFISH) images. This deep learning model effectively reduced human bias and improved the automation of CTC detection. Faced with the problem of complex CTC morphology and limited classification accuracy of a single method, a CNN-SVM hybrid method was proposed [59], using CNN for feature extraction and SVM for classification. The algorithm classified CTC clusters of various configurations with >90% sensitivity and specificity. Facing the challenge of accurately identifying CTCs amidst a vast number of blood cells, Wei et al. [75] employed a random forest classifier that focuses on the geometric shapes and brightness variations of cells in blood images. This ML-based approach facilitated the precise detection of CTCs, achieving high accuracy and demonstrating the effectiveness of the method over conventional techniques. Table 2 summarizes the applications of ML algorithms in the analysis of CTCs.

**Figure 4:**
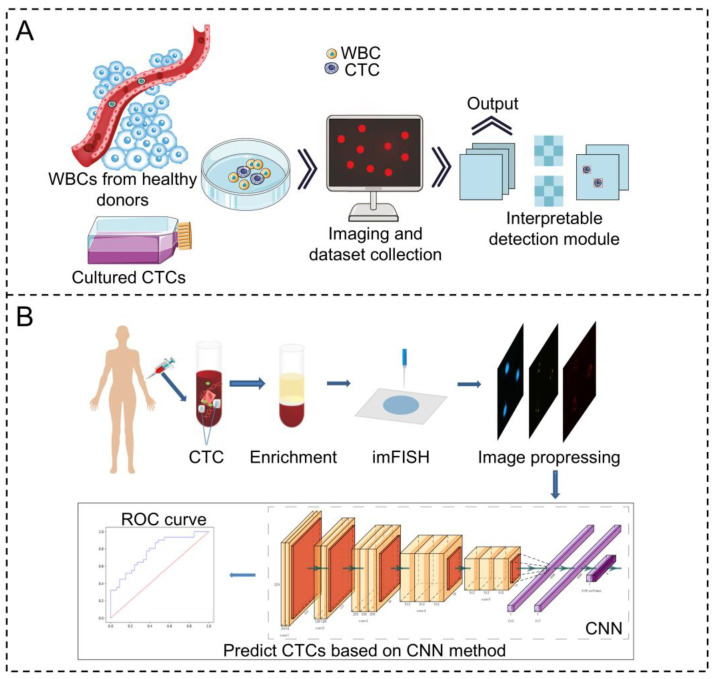
(**A**) Schematic illustration for the interpretatively automated identification of CTCs in spiked samples, where CTCs are added in human peripheral blood containing WBCs. Reproduced with permission from [82]. (**B**) The complete process of identifying CTCs based on CNN. First, peripheral blood of tumor patients is collected, and then the blood samples are processed. Based on immunological principles, CTCs are enriched by gradual removal of plasma, red blood cell, and white blood cell components with the help of magnetic particle technology, and CTCs are treated by imFISH. Finally, after the image is preprocessed, stratified sampling is used to segment the data, and the deep learning algorithm based on CNN is used to train the model. Reproduced with permission from [25].

To achieve high-accuracy, fully automatic detection, numerous ML methods have been proposed for CTC detection. Figure 4A shows a schematic diagram of the automatic identification of CTCs. Svensson et al. [76] introduced a naive Bayes classifier that utilizes features extracted from relevant image areas for detecting CTCs. This classifier successfully detected and counted CTCs in fluorescence images. However, this method could not overcome the limitations of complex and variable smear environments, uneven illumination, uneven staining, cell adhesion, and environmental contaminants, leading to a high false-positive rate and poor practicality. Mao and colleagues [77] designed a deep convolutional network with automatic feature learning for image-based CTC detection. Their findings demonstrated that deep learning methods outperformed ML by recognizing specific CTCs mixed with red blood cells (mixing ratio of 1:10,000). Mocan [78] utilized an improved U-Net capable of automatic image segmentation for CTC detection. Ciurte et al. [79] proposed a CNN method enhanced by an Adaboost classifier, achieving a total sensitivity of 92.87% and a specificity of 99.98% for CTC detection, comparable to human expert detection results. Nissim and others [80] employed ML techniques (SVM classifier) to process acquired images and classify cancer cells and four types of blood cells based on their morphology and quantitative phase features. This method achieved a high accuracy of 92.56% in distinguishing between different cell types, enabling further automatic enrichment and cancer cell grading in the blood. Zeune et al. [81] compared standard CNNs with autoencoder CNNs, combining autoencoder CNNs with advanced visualization techniques to categorize single-cell fluorescence images into five different classes, achieving accuracies, sensitivities, and specificities exceeding 96%, and the obtained CTC counts predicted overall survival in cancer patients as well as the most advanced manual counting. In the study by Guo et al. [25] (Figure 4B), a novel convolutional neural network (CNN) approach was developed to automatically detect CTCs in patients’ peripheral blood based on immunofluorescence in situ hybridization (imFISH) images. Sensitivity and specificity based on traditional CNN predictions were 95.3% and 91.7%, respectively, while those based on transfer learning were 97.2% and 94.0%, respectively, offering clinical reference value for prognosis judgment and metastasis diagnosis.

**Figure 5:**
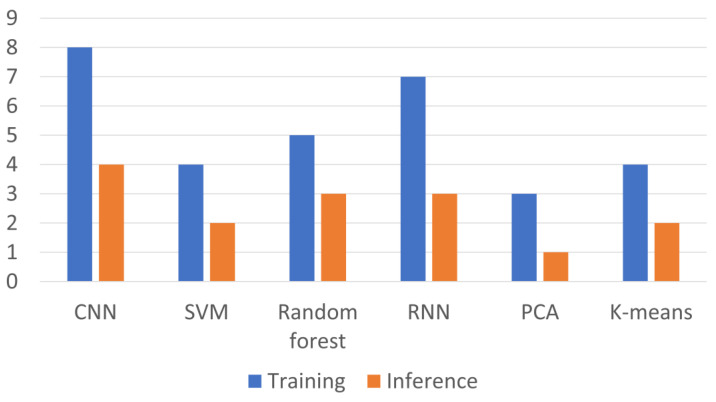
Comparison of computational resource consumption of different ML algorithms. The chart compares several ML models in terms of computational resource consumption during the training and inference phases. In the bar chart, blue represents resource usage during training, and orange represents usage during inference (i.e., model deployment).

In the detection of CTCs, different ML models demonstrate varied applications, each with its computational resource implications during training and inference stages. Figure 5 illustrates the computational resource consumption of different ML algorithms during training and inference phases. CNN show exceptional image recognition capabilities for CTCs, yet they are resource-intensive during the training phase, requiring significant computational power. Despite their high training demands, they become considerably more efficient in the inference stage, allowing for rapid and accurate classification of CTCs in practical applications [25]. Support vector machines (SVMs), known for their power in classifying well-defined features, exhibit a moderate computational load during training and are notably efficient during inference. This makes SVMs an attractive option for real-time CTC detection where computational resources might be limited [80]. Random forests, which process a multitude of features through multiple decision trees, show an intermediate level of computational consumption in both training and inference phases. Their balanced approach is suitable for datasets with complex characteristics, like the varied morphology of CTCs [59]. Recurrent neural networks (RNNs) are tailored for sequential data analysis, such as tracking the dynamic behavior of CTCs over time. They align with CNNs in terms of higher computational usage during the training period but offer the advantage of capturing temporal changes during CTC analysis [76]. Principal component analysis (PCA) serves as a method for dimensionality reduction, simplifying the ML processing flow with minimal computational demands, especially during inference, thereby providing a streamlined approach for highlighting significant features within CTC datasets [77]. Lastly, unsupervised learning models like clustering algorithms, for instance, K-means, are useful for discovering various cell populations with less computational stress compared to deep learning models, making them suitable for initial explorative analysis of CTCs without the need for labeled data [83].

## 4. Integration of ML and Microfluidics for Early Cancer Detection Through CTCs

### 4.1. Fundamentals of Tumor Analysis

Tumor analysis plays a pivotal role in the early detection of cancer, as it helps to identify the molecular and genetic alterations that occur in the early stages of tumor development [84,85]. Cancer is often most treatable when detected early, but early-stage tumors are frequently asymptomatic and difficult to detect using conventional methods like imaging. Therefore, tumor analysis aims to uncover early molecular markers and biomarkers that can be detected in body fluids, enabling non-invasive diagnostic techniques. For example, the identification of genetic mutations, such as those in the EGFR gene for lung cancer or BRCA1/2 for breast cancer, can provide early signals of cancer risk or the presence of cancer at a molecular level, often before physical symptoms appear.

Molecular profiling of tumors involves examining the genetic, transcriptomic, and proteomic alterations in tumor cells [86,87]. Advanced techniques like next-generation sequencing (NGS) and liquid biopsy, which detects tumor-derived material such as circulating tumor DNA (ctDNA) or CTCs in blood samples, have revolutionized early cancer detection. These techniques allow for the identification of cancer-related mutations, gene fusions, or abnormal protein expressions that serve as early indicators of malignancy. Liquid biopsy is especially promising for early cancer detection, as it provides a non-invasive way to monitor cancer’s genetic profile and detect the presence of tumor cells in the bloodstream long before physical symptoms or tumors become detectable by imaging.

Histopathological analysis, though often used in later stages of cancer diagnosis, can also play a role in early detection by providing detailed insights into the morphology of cells. Advances in staining techniques and digital pathology have improved the ability to detect abnormal cellular growth patterns and micro-metastases at an early stage. The integration of these molecular, genetic, and histological analyses provides a comprehensive approach to early cancer diagnosis, facilitating the identification of tumors at a stage when they are more treatable and before they have spread, ultimately improving patient outcomes and survival rates.

### 4.2. Integrating ML and Microfluidics for Early Cancer Detection Through CTCs

**Figure 6:**
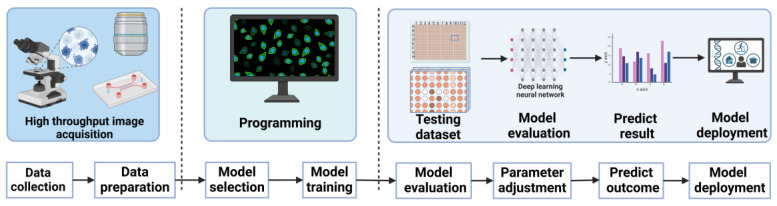
Schematic of the workflow for high-throughput biological data analysis integrating ML with microfluidic technology. The workflow combining ML with microfluidic technology begins with the efficient collection of biological data using microfluidics, followed by data preprocessing and the selection and programming of ML models. This is then followed by model training, testing, and evaluation. Finally, parameter adjustment and outcome prediction are carried out to optimize model performance, and the model is deployed in practical applications for the automated processing and analysis of high-throughput biological data. (Created with BioRender.com).

The integration of artificial intelligence and microfluidic technologies is emerging as an attractive research theme in the scientific community for addressing open biomedical problems [88,89,90]. Particularly in recent years, the convergence of ML and microfluidic technologies has brought revolutionary advancements to the detection and analysis of CTCs, which is critically important for the early diagnosis and treatment monitoring of cancer. Microfluidic technologies enable the precise control of fluid flow within micro-channels, effectively capturing and isolating minute quantities of CTCs and allowing for meticulous manipulation at the single-cell level, thus enabling in-depth research [91]. The application of ML is particularly crucial in data processing and cell recognition. Researchers can utilize ML algorithms to extract valuable information from complex datasets, such as the morphology, size, and biomarker expression of cells. These algorithms are capable of learning to differentiate between CTCs and non-tumor cells and can even identify subgroups among CTCs, thereby enhancing the accuracy and efficiency of detection [25]. Furthermore, deep learning models within ML can analyze historical data to predict the behavioral patterns and potential variations of CTCs, offering more precise disease monitoring and management strategies for clinical use. ML also plays a significant role in optimizing the design and operation of microfluidic devices. By simulating different fluid dynamics conditions and cell behaviors, ML models can assist engineers in designing more effective CTC capture systems [92,93]. These improvements in system design, combined with real-time data analysis, significantly enhance the sensitivity and specificity of CTC detection. The development of this integrated technology not only boosts the processing capabilities for biological samples but also drives the implementation of personalized medicine and precision treatment strategies, offering unprecedented hope and potential for cancer treatment. Figure 6 illustrates the workflow for high-throughput biological data analysis, integrating ML with microfluidic technology for automated data processing and analysis.

Integrating ML with microfluidic systems has significantly enhanced the detection and analysis of CTCs by improving data acquisition, real-time processing, and decision-making processes. In terms of data acquisition, Microfluidic devices enable the efficient isolation and enrichment of CTCs from blood samples. For instance, a study developed a hyperuniform, micropost, microfluidic device that captures the trajectories of CTCs. By analyzing these trajectories, ML models can classify different CTC phenotypes, facilitating early cancer detection [94]. In terms of real-time processing, the integration of ML algorithms with microfluidic platforms allows for the real-time analysis of acquired data. In one study, a deep-learning-based method was developed to automatically identify tumor cells mimicking CTCs and cancer-associated fibroblasts (CAFs). This approach enabled real-time monitoring and classification of CTCs during flow cytometry analysis [91]. In terms of decision-making processes, ML algorithms enhance decision-making by providing predictive analytics based on analyzed data. For example, a study addressed the challenges in distinguishing CTCs from non-CTCs in cancer patient blood samples by introducing a human-in-the-loop (HiL) strategy. This method integrates self-supervised deep learning with a conventional ML-based classifier, allowing iterative targeted sampling and expert labeling of uncertain cases. By refining the training dataset and improving classification accuracy, this approach enhances the reliability of ML-driven CTC detection, ultimately supporting more accurate and informed clinical decisions in patient management [56].

**Figure 7:**
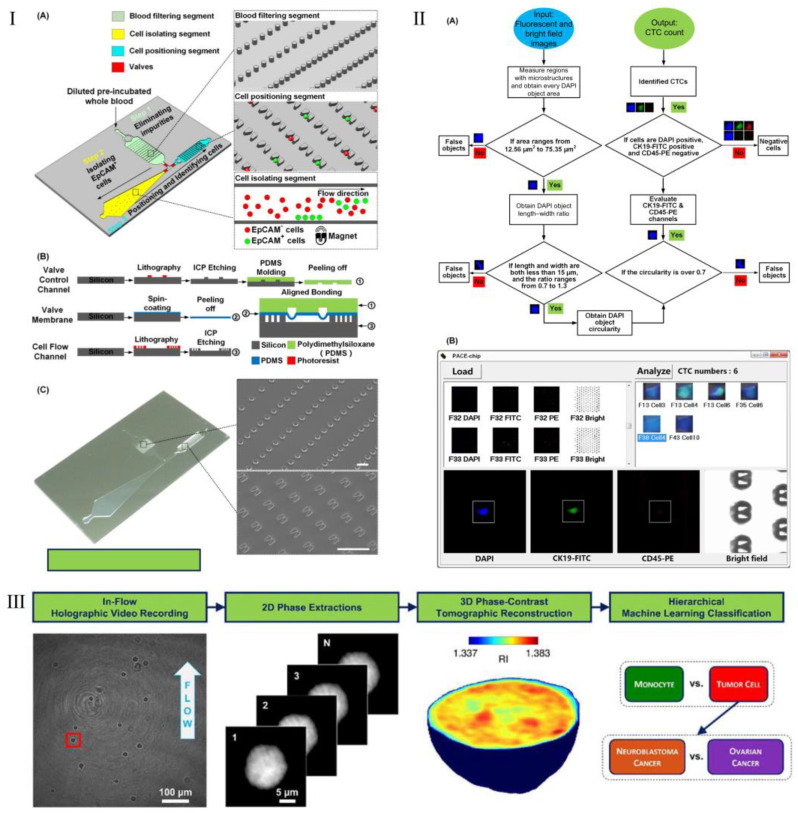
Examples of the integration of ML and microfluidics in the detection of CTCs. (**I**,**II**) A microfluidic platform composed of a multifunctional microfluidic chip and a unique image processing algorithm. (**I**) The integrated microfluidic design of the PACE-chip. (**A**) Schematic illustration of the PACE-chip, which is composed of three functional regions: blood filtration (top-right inset shows the micro-pillar array), cell isolation (bottom-right inset highlights the EpCAM-positive cell selection process), and cell positioning (middle-right inset displays the V-shaped microstructure array). (**B**) Workflow of the PACE-chip fabrication process. (**C**) Photomicrographs of the silicon-based PACE-chip, including detailed SEM images of the micro-pillar structures (top-right) and the V-shaped microstructures (bottom-right). Scale bars: 100 µm. (**II**) Computer-aided image processing. (**A**) Diagram illustrating the workflow of the image processing algorithm. (**B**) Screenshot of the user interface running on a Windows™ operating system. Reproduced with permission from [95]. (**III**) Steps of the ML-powered tomographic phase imaging flow cytometry. Reproduced with permission from [96].

In recent years, the rapid and automated identification of CTCs has become increasingly important, and research into automated processes for CTC recognition has accelerated. Figure 7 illustrates examples of the integration of ML and microfluidic technologies in the detection of CTCs. For instance, Zhou et al. [95] developed a microfluidic platform composed of a multifunctional microfluidic chip, an automated imaging device, and a unique image processing algorithm. This microfluidic chip integrates blood filtering, cell separation, and single-cell positioning to ensure minimal cell loss, efficient cell separation, and fixed arraying of single cells to facilitate downstream image processing. Automated fluorescence image acquisition and processing replace subjective manual interpretation with an objective criterion. By leveraging the design of the microfluidic chip to reduce computational load and eliminate measurement errors, the specially designed algorithm is capable of rapidly interpreting hundreds of images to provide accurate CTC counts. After intensive optimization of the microfluidic chip, the image processing algorithm, and their collaboration, the complete platform was validated by enumerating CTCs from six clinical blood samples of breast cancer patients. Compared to tube-based CTC isolation and manual CTC identification, the microfluidic platform demonstrated higher accuracy and reduced the time required by 50%. This automated CTC enumeration platform not only presents a rational strategy by integrating a specially designed multifunctional microfluidic chip with a unique image processing algorithm for robust, accurate CTC enumeration but may also lead to its use as a novel in vitro diagnostic device, as readily applicable in clinics and laboratories as a routine blood test. Pirone et al. [96] proposed a new AI-driven method for CTCs detection that records digital holograms of individual cells while they flow and rotate along a microfluidic channel, and by retrieving their 2D refractive index maps, a dataset of 3D RI tomographic images is collected for analysis by a tiered ML decision-maker. In the first stage, it differentiates leukocytes from tumor cells, followed by tumor type recognition at a second decision level. The study demonstrated that this label-free method could rapidly and effectively perform phenotypic analysis of cancer cells, serving as a suitable tool for identifying new morphological biomarkers to differentiate CTCs and distinguish clinically aggressive CTCs from more favorable ones. Gangadhar et al. [97] used digital holographic microscopy (DHM), microfluidics, and ML for the label-free enumeration of tumor cells against a background of white blood cells, proposing a method that could offer a rapid screening tool for the label-free enumeration of CTCs.

ML not only integrates with microfluidic technology to enhance the detection accuracy of CTCs, but it also simplifies the design of microfluidic systems by predicting the impacts of various design parameters on performance, thereby increasing efficiency and reducing costs [98]. It also enables precise control over fluid dynamics in real time, adjusting to changes in flow rate, pressure, and temperature based on experimental needs. For experiment optimization, ML analyzes historical data to identify optimal conditions, reducing the resources and time needed for development. Additionally, it adeptly handles and interprets the growing volume and complexity of data in microfluidics, extracting key insights that drive further innovations and applications in the field.

### 4.3. Recent Advancements in Early Cancer Detection Technologies

Early detection is crucial in cancer treatment, significantly improving patient survival rates. Recent advancements in microfluidic technologies and ML have led to innovative diagnostic methods that facilitate the early identification of various cancers [99,100]. Microfluidic technologies, which involve the precise control and manipulation of fluids at the microscale, have been instrumental in developing point-of-care testing (POCT) devices for early cancer detection. These devices can process small volumes of biological samples, such as blood, saliva, or urine, to detect cancer biomarkers efficiently. For instance, microfluidic chips have been designed to capture CTCs and cell-free DNA (cfDNA), enabling real-time analysis without the need for complex laboratory setups. This capability allows for rapid and accurate detection of cancer-related biomarkers, facilitating early diagnosis and timely intervention. Similarly, advancements in microfluidic chip technology have significantly improved the detection of lung cancer. These chips can capture exosomes from plasma, which are small vesicles released by cells containing tumor-specific molecules [101,102]. The integration of microfluidic chips with exosome analysis has led to a tenfold increase in detection speed and a fourteenfold enhancement in sensitivity, greatly improving the prospects for early diagnosis. 

The incorporation of ML algorithms into diagnostic processes has further revolutionized early cancer detection. ML models can analyze complex datasets, identify patterns, and make predictions that aid in diagnosis and treatment planning. For example, a study demonstrated the use of interferometric phase microscopy combined with ML for real-time, label-free classification of cancer cells and blood cells. This method achieved a high accuracy rate of 92.56% in distinguishing between different cell types, paving the way for automatic cancer cell classification in blood samples [81]. Furthermore, the development of AI-driven projection tomography systems has enabled three-dimensional imaging of cancer cells, providing detailed morphological information that enhances early detection capabilities. These systems utilize fiber-optic cell rotation and AI algorithms to reconstruct high-resolution images of cells, facilitating the identification of malignant transformations at an early stage. The convergence of microfluidic technologies and ML has led to the development of advanced diagnostic platforms that offer rapid, accurate, and minimally invasive cancer detection. For instance, microfluidic devices integrated with ML algorithms can process and analyze biological samples in real time, enabling the detection of subtle changes associated with early-stage cancers. This integration enhances the sensitivity and specificity of diagnostic tests, leading to improved patient outcomes. Table 3 summarizes recent advancements in early cancer detection technologies, highlighting innovations in microfluidics and machine learning across various cancer types.

## 5. Challenges and Limitations

### 5.1. Practical Considerations and Challenges

While ML algorithms have demonstrated significant promise in advancing CTC detection, their practical implementation in clinical settings presents several challenges.

One major concern is the computational resource demand associated with these algorithms. For instance, deep learning models such as CNNs and transformer-based architectures require large-scale datasets and high-performance GPUs or cloud computing resources to function effectively. In a real-world clinical setting, many hospitals and diagnostic laboratories, particularly in low-resource regions, lack the computational infrastructure to deploy and maintain such models efficiently. This limitation can hinder the widespread adoption of AI-driven CTC detection systems outside of research laboratories or well-funded medical institutions.

Additionally, economic feasibility remains a key barrier. The cost of AI-driven CTC detection goes beyond initial algorithm development; it includes data collection, annotation, model validation, regulatory approvals, and integration into existing diagnostic workflows. For example, a deep-learning-based CTC classification system trained on high-resolution imaging data may require thousands of manually labeled samples from multiple institutions, significantly increasing costs. Furthermore, commercial microfluidic platforms incorporating AI-driven detection, must justify their cost-effectiveness compared to traditional immunocapture-based approaches before gaining clinical acceptance. Without clear evidence that ML-based methods offer superior accuracy, cost savings, or faster turnaround times, their adoption may be slow in routine clinical practice.

Another challenge is clinical translation and regulatory approval. While AI-driven CTC detection models can achieve high sensitivity and specificity in controlled research settings, their performance in real-world clinical applications may vary due to heterogeneous patient data, differences in sample preparation techniques, and variability in imaging or sensor technology across laboratories. The FDA’s approval process for AI-based diagnostic tools requires extensive validation studies across diverse patient populations, adding to the timeline and cost of clinical implementation. For example, the CellSearch^®^ system, an FDA-approved CTC detection platform, underwent rigorous multicenter validation before clinical adoption. ML-based alternatives would require similar, if not greater, validation efforts to gain regulatory acceptance.

Moreover, scalability and integration into clinical workflows present significant hurdles. Many ML models operate as standalone tools requiring specialized expertise for interpretation. For AI-driven CTC detection to be clinically viable, it must seamlessly integrate into existing electronic health record (EHR) systems, laboratory information systems (LIS), and automated microfluidic workflows. Without a user-friendly interface and automated decision support, clinicians may be hesitant to rely on ML-based predictions for patient management.

### 5.2. Ethical and Privacy Considerations

In the realm of microfluidic applications for detecting CTCs, the integration of ML introduces several ethical and privacy concerns that must be addressed to ensure responsible and fair implementation in clinical settings. Data privacy and security are paramount, as ML-driven CTC detection relies on large datasets containing sensitive patient health information. Without stringent safeguards, there is a risk of unauthorized access, data breaches, or misuse of patient records, which could undermine trust in AI-powered diagnostic systems. Compliance with data protection regulations such as HIPAA (Health Insurance Portability and Accountability Act) and GDPR (General Data Protection Regulation) is essential to ensure that patient confidentiality is maintained throughout the data collection, processing, and model deployment phases. Techniques such as federated learning, differential privacy, and secure multi-party computation can enhance data protection while allowing model training on decentralized datasets.

Beyond privacy concerns, algorithmic fairness and bias pose significant ethical challenges in CTC detection. ML models trained on unrepresentative or biased datasets may lead to disparities in diagnostic accuracy across different patient demographics. For instance, if training data predominantly originate from a specific population group, the model may exhibit reduced sensitivity when applied to underrepresented ethnicities, age groups, or cancer subtypes. This could result in misclassifications or unequal access to early cancer diagnosis, reinforcing existing healthcare disparities. Addressing this issue requires systematic efforts to ensure diverse and unbiased data collection, transparent feature selection, and the use of fairness-aware algorithms that mitigate unintended biases. Rigorous external validation on heterogeneous patient cohorts should be a mandatory step before deploying ML-based CTC detection tools in real-world clinical environments.

Additionally, regulatory approval processes for AI-driven diagnostic tools remain a major hurdle. Unlike traditional CTC detection methods, ML models introduce dynamic decision-making and continuous learning, which complicates standard regulatory frameworks. Regulatory bodies such as the FDA and EMA (European Medicines Agency) require robust validation, explainability, and reproducibility of AI models before they can be integrated into clinical workflows. However, many deep learning models function as “black boxes”, making it difficult for clinicians and regulators to interpret their decision-making processes. Implementing explainable AI (XAI) techniques can improve transparency, allowing healthcare professionals to understand how an ML model arrives at its predictions.

## 6. Discussion

To provide a deeper understanding of the integrated technologies and their impact on early cancer detection, we now present a SWOT analysis evaluating the strengths, weaknesses, opportunities, and challenges of this review: The primary strength of this review is the comprehensive nature of the topic. The article simultaneously introduces microfluidic technology, machine learning, and their integration for CTC detection, highlighting the importance of this fusion in early cancer diagnosis. By focusing on these cutting-edge technologies, the review addresses a highly relevant and innovative area of cancer research that has the potential to significantly improve early detection and personalized treatment approaches. One potential weakness of the review is the complexity of integrating two advanced technologies, which may limit its appeal to a broader audience. While the review is highly informative for experts in the fields of microfluidics, machine learning, and cancer diagnostics, it might be difficult for those without a deep technical background to fully appreciate the nuances of the technologies discussed. As the demand for early cancer detection continues to rise, there is great potential for the integration of microfluidics and machine learning to play an even larger role in clinical practice. Future research could explore the development of more standardized, user-friendly systems for cancer detection that integrate these technologies more seamlessly into routine medical practices. Despite the potential of this integrated approach, there are threats that could hinder its widespread adoption. One significant challenge is the cost and technical complexity of implementing microfluidic systems combined with machine learning algorithms in clinical settings. The development of cost-effective and scalable solutions will be crucial for these technologies to gain widespread acceptance. Additionally, competition from other diagnostic methods, such as imaging techniques and traditional biomarkers, may limit the adoption of these novel technologies.

## 7. Conclusions

In conclusion, the integration of ML algorithms with microfluidic technologies has significantly advanced the field of early cancer diagnosis, particularly through the detection of CTCs. This review highlights the remarkable progress made in both microfluidic systems and machine learning, showcasing how their combination enhances the specificity, accuracy, and efficiency of CTC detection. Microfluidic platforms provide a powerful tool for isolating and analyzing CTCs from blood samples, offering a non-invasive alternative to traditional biopsy methods. Coupled with machine learning, these systems can automate complex data processing and analysis, improving the ability to detect rare and heterogeneous tumor cells. As a result, this integration holds great promise in enabling early detection, monitoring cancer progression, and developing personalized treatment strategies. While challenges remain, such as standardization, cost, and technical complexity, the continuous development of both technologies offers significant potential for revolutionizing cancer diagnostics.

## Figures and Tables

**Table 1:** Comparison of CTC isolation technologies: advantages, limitations, application scenarios, and performance metrics.

Technology Category	Specific Technology	Advantages	Disadvantages	Application Scenarios	Efficiency	Sensitivity	Specificity	Throughput	Cell Viability
Physical (Size-Based) Methods	Microfiltration	High throughput, low cost, suitable for large sample volumes	Risk of clogging; may cause cell deformation	Pre-treatment of blood, bulk initial separation	High	Moderate	Moderate	High	Moderate
	Inertial Focusing	Label-free, rapid processing	Lower separation purity; may require further refinement	Continuous flow separation, rapid screening	High	High	Moderate	High	High
Affinity-Based Methods	Immunocapture	High specificity and selectivity; direct use for downstream analysis	Dependent on known CTC surface markers; lower throughput	Precise target cell capture, subsequent molecular analysis	Moderate	Very High	Very High	Moderate	Moderate
	Magnetic Bead Separation	High specificity; adjustable binding efficiency	Requires magnetic labeling; involves multiple processing steps	Clinical diagnostics, targeted enrichment	Moderate	High	Very High	Low	Moderate
Electrical-Based Methods	Dielectrophoresis	High precision, label-free, tunable	High equipment cost; complex operation and integration	Research applications, separation based on electrical properties	Moderate	High	High	Low	High
Integrated/Multi-Modal Platforms	CTC-iChip	Combines multiple separation principles; comprehensive performance; high automation	Complex design; higher initial cost	Precision medicine, comprehensive cell analysis	High	High	High	High	Moderate

**Table 2:** Application of ML algorithms in CTCs analysis.

Method	Function	Advantages	Limitations	Application Scenarios
CNN	Extracts hierarchical spatial features from image data, enabling automated feature learning for CTC recognition	Efficient image recognition and classification capability, suitable for identifying specific morphologies and markers of CTCs	Requires large, labeled datasets for training, computationally expensive	Recognition and classification of CTCs in microfluidic chip images
SVM	Maps data into a high-dimensional space to find the optimal hyperplane for classifying CTCs	Powerful classification performance, suitable for classification problems with clear boundaries	Sensitive to noisy data and outliers, less effective with unbalanced datasets	Differentiating CTCs from other blood cells, selective classification of cell features
Random Forest	Uses an ensemble of decision trees to improve classification reliability and handle complex, high-dimensional data	Improves accuracy and robustness by constructing multiple decision trees, suitable for handling a large number of features	Can overfit with noisy data, less interpretable than simpler models	Processing a large number of features, such as cell morphology and molecular markers, to improve classification accuracy
RNN	Captures temporal dependencies in sequential data, enabling dynamic tracking of CTC behaviors	Capability to process sequential data, useful for analyzing dynamic changes of CTCs	Struggles with very long sequences, requires large datasets for training	Monitoring the behavior of CTCs in microfluidic devices
PCA	Transforms high-dimensional data into a reduced set of principal components, preserving the most significant variance	Data dimensionality reduction, highlighting important features, simplifying the machine learning processing flow	May lose some important variance during dimensionality reduction	Reducing the dimensionality of CTC image data, highlighting the most important features
K-means	Partitions data into clusters based on similarity, helping to classify and differentiate CTC subpopulations	Unsupervised learning, can automatically discover different cell populations	Sensitive to the initial choice of K, ineffective with clusters of varying sizes	Automatically discovering different cell populations based on cell morphology and phenotypic features

**Table 3:** Recent advancements in early cancer-detection technologies.

Technology	Description	Cancer Type	Advantages	Reference
Trajectory-Based ML Classification in Microfluidics	Classifies CTCs by analyzing their movement trajectories in microchannels using ML trained on simulation-based datasets.	General (validation study)	Label-free, interpretable trajectory data, combines simulation and ML.	[103]
Automated Microfluidic HER2-CTC Analysis	Enumerates CTCs and evaluates HER2 expression via an automated microfluidic system for breast cancer prognosis.	Metastatic breast dancer	Automated, prognostic relevance, HER2 marker integration.	[104]
Deep Learning + Label-Free Flow Cytometry	Uses deep learning to detect rare CTC clusters in whole blood through label-free flow cytometry.	General	Label-free, detects rare clusters, high accuracy with DL.	[105]
Inertial Microfluidics for Pancreatic CTCs	Enriches portal vein CTCs in CA19-9-negative pancreatic cancer patients using inertial flow-based separation.	Pancreatic cancer	High throughput, useful for biomarker-negative cases.	[106]
Patient-Derived Microfluidic Disease Chips	Integrates microfluidic disease models with deep learning for precision diagnostics on patient-derived samples.	General (patient-specific)	Patient-specific, integrative ML platform, personalized.	[107]
Tomographic Phase Imaging + ML	Combines tomographic phase imaging with ML for label-free CTC detection in liquid biopsy.	General	Label-free, optical imaging + ML, early detection.	[108]
Biolaser + Deep Learning CTC Detection	Uses DL-enhanced biolaser readouts for antigen-independent single-cell CTC detection.	General	Antigen-independent, single-cell precision, DL-powered.	[109]
Ultrasound-Assisted Microfluidic Separation	Studies microparticle dynamics in ultrasound-enhanced microfluidics for potential cancer diagnostics.	General/exploratory	Non-contact, gentle separation, tunable system.	[110]
Microfluidic Biosensors for Body Fluid Biomarkers	Reviews microfluidic biosensors for detecting cancer biomarkers in fluids like blood, urine, and saliva.	Various (review)	Non-invasive, biosensor integration, early diagnosis.	[111]
AFM + Microfluidics for CTC Nano-Mechanics	Uses atomic force microscopy in microfluidic chips to sort and analyze nanomechanical properties of CTCs.	General	Label-free, mechanical profiling, single-cell level.	[112]

## References

[1] Li C., He W., Wang N., Xi Z., Deng R., Liu X., Kang R., Xie L., Liu X. (2022). Application of microfluidics in detection of circulating tumor cells. Front. Bioeng. Biotechnol..

[2] Wild C.P., Weiderpass E., Stewart B.W. (2020). World Cancer Report.

[3] Cai J., Chen B., He M., Yuan G., Hu B. (2024). An Integrated Inertial-Magnetophoresis Microfluidic Chip Online-Coupled with ICP-MS for Rapid Separation and Precise Detection of Circulating Tumor Cells. Anal. Chem..

[4] Lyu N., Hassanzadeh-Barforoushi A., Rey Gomez L.M., Zhang W., Wang Y. (2024). SERS biosensors for liquid biopsy towards cancer diagnosis by detection of various circulating biomarkers: Current progress and perspectives. Nano Converg..

[5] Li L., Jiang H., Zeng B., Wang X., Bao Y., Chen C., Ma L., Yuan J. (2024). Liquid biopsy in lung cancer. Clin. Chim. Acta.

[6] Chen J., Liu C.-Y., Wang X., Sweet E., Liu N., Gong X., Lin L. (2020). 3D printed microfluidic devices for circulating tumor cells (CTCs) isolation. Biosens. Bioelectron..

[7] Lao Z., Ren X., Zhuang D., Xie L., Zhang Y., Li W., Chen Y., Li P., Tong L., Chu P.K. (2025). A phenotype-independent “label-capture-release” process for isolating viable circulating tumor cells in real-time drug susceptibility testing. Innovation.

[8] Shi J., Zhang H., Cui Y., Xing J., Wang W., Chen J., Wang S., Yang Z. (2024). Extracellular vesicles for breast cancer diagnosis and therapy. Extracell. Vesicle.

[9] Ashworth T. (1869). A case of cancer in which cells similar to those in the tumours were seen in the blood after death. Aust. Med. J..

[10] Williams J., Sanchez S., Hernandez M. (2024). Research on the biophysical properties of circulating tumor cells. Camb. Sci. Adv..

[11] Shi R., Yue Y., Liu Z., Chai H., Miao P. (2024). Recent advances in integrated biophysical and biochemical microfluidic methods for circulating tumor cells isolation and analysis. Fundam. Res..

[12] Li W., Guo Z., Zhou Z., Zhou Z., He H., Sun J., Zhou X., Chin Y.R., Zhang L., Yang M. (2024). Distinguishing high-metastasis-potential circulating tumor cells through fluidic shear stress in a bloodstream-like microfluidic circulatory system. Oncogene.

[13] Tukova A., Zhang W., Rodger A., Wang Y. (2024). Analytical SERS for Liquid Biopsy Biomarkers Detection. Surface and Tip-Enhanced Raman Scattering Spectroscopy: Bridging Theory and Applications.

[14] Abusamra S.M., Barber R., Sharafeldin M., Edwards C.M., Davis J.J. (2024). The integrated on-chip isolation and detection of circulating tumour cells. Sens. Diagn..

[15] Lin D., Shen L., Luo M., Zhang K., Li J., Yang Q., Zhu F., Zhou D., Zheng S., Chen Y. (2021). Circulating tumor cells: Biology and clinical significance. Signal Transduct. Target. Ther..

[16] Bagheri R., Ghorbian M., Ghorbian S. (2024). Tumor circulating biomarkers in colorectal cancer. Cancer Treat. Res. Commun..

[17] Bu J., Lee T.H., Poellmann M.J., Rawding P.A., Jeong W.J., Hong R.S., Hyun S.H., Eun H.S., Hong S. (2021). Tri-modal liquid biopsy: Combinational analysis of circulating tumor cells, exosomes, and cell-free DNA using machine learning algorithm. Clin. Transl. Med..

[18] Zhang C., Xu L., Miao X., Zhang D., Xie Y., Hu Y., Zhang Z., Wang X., Wu X., Liu Z. (2025). Machine learning assisted dual-modal SERS detection for circulating tumor cells. Biosens. Bioelectron..

[19] Wang Y., Cao N., Cui X., Liu Z., Yuan X., Chen S., Xu H., Yi M., Ti Y., Zheng F. (2024). Detection of circulating tumor cells using a microfluidic chip for diagnostics and therapeutic prediction in mediastinal neuroblastoma. Eur. J. Pediatr..

[20] Kilercik M., Özgür E., Şahin Ş., Şen Doğan B., Mutlu E., Cihan C., Kolay M., Erkal N., Zorlu Ö., Doğanca T.S. (2024). Detection of circulating tumor cells in non-metastatic prostate cancer through integration of a microfluidic CTC enrichment system and multiparametric flow cytometry. PLoS ONE.

[21] Jiang Y., Yang M., Wang S., Li X., Sun Y. (2020). Emerging role of deep learning-based artificial intelligence in tumor pathology. Cancer Commun..

[22] Douglass P.M., O’Connor T., Javidi B. (2022). Automated sickle cell disease identification in human red blood cells using a lensless single random phase encoding biosensor and convolutional neural networks. Opt. Express.

[23] Pasupa K., Vatathanavaro S., Tungjitnob S. (2023). Convolutional neural networks based focal loss for class imbalance problem: A case study of canine red blood cells morphology classification. J. Ambient Intell. Humaniz. Comput..

[24] Wang S., Zhou Y., Qin X., Nair S., Huang X., Liu Y. (2020). Label-free detection of rare circulating tumor cells by image analysis and machine learning. Sci. Rep..

[25] Guo Z., Lin X., Hui Y., Wang J., Zhang Q., Kong F. (2022). Circulating tumor cell identification based on deep learning. Front. Oncol..

[26] Liu Y., Zhao W., Hodgson J., Egan M., Cooper Pope C.N., Hicks G., Nikolinakos P.G., Mao L. (2024). CTC-race: Single-cell motility assay of circulating tumor cells from metastatic lung cancer patients. ACS Nano.

[27] Kulkarni M.B., Goel S. (2020). Microfluidic devices for synthesizing nanomaterials—A review. Nano Express.

[28] Huang Y., Liu C., Feng Q., Sun J. (2023). Microfluidic synthesis of nanomaterials for biomedical applications. Nanoscale Horiz..

[29] Elvira K.S., Gielen F., Tsai S.S., Nightingale A.M. (2022). Materials and methods for droplet microfluidic device fabrication. Lab Chip.

[30] Hua X., Liu X., Zhu Q., Liu Y., Zhou S., Huang P., Li Q., Liu S. (2022). Three-dimensional microfluidic chip for efficient capture of secretory autophagosomes and sensitive detection of their surface proteins. Anal. Chem..

[31] Battat S., Weitz D.A., Whitesides G.M. (2022). Nonlinear phenomena in microfluidics. Chem. Rev..

[32] Wu L., Guo Z., Liu W. (2022). Surface behaviors of droplet manipulation in microfluidics devices. Adv. Colloid Interface Sci..

[33] Kronfeld K.-P., Köhler J.M., Ellinger T. (2024). Microfluidic synthesis and properties of thermoresponsive hydrogel core–shell particles. J. Compos. Sci..

[34] Rahmanizadeh M. (2022). Design and Fabrication of Thermo-Responsive Hydrogel Particles with Tunable Swelling and Mechanical Behavior via Microfluidics Method. https://www.politesi.polimi.it/handle/10589/219778.

[35] Azizipour N., Avazpour R., Sawan M., Rosenzweig D.H., Ajji A. (2022). Uniformity of spheroids-on-a-chip by surface treatment of PDMS microfluidic platforms. Sens. Diagn..

[36] Ramasamy M., Ho B., Phan C.-M., Qin N., Ren C.L., Jones L. (2023). Inexpensive and rapid fabrication of PDMS microfluidic devices for biological testing applications using low cost commercially available 3D printers. J. Micromech. Microeng..

[37] McIntyre D., Lashkaripour A., Fordyce P., Densmore D. (2022). Machine learning for microfluidic design and control. Lab Chip.

[38] Lashkaripour A., McIntyre D.P., Calhoun S.G., Krauth K., Densmore D.M., Fordyce P.M. (2024). Design automation of microfluidic single and double emulsion droplets with machine learning. Nat. Commun..

[39] Ahmed M.G., Abate M.F., Song Y., Zhu Z., Yan F., Xu Y., Wang X., Li Q., Yang C. (2017). Isolation, detection, and antigen-based profiling of circulating tumor cells using a size-dictated immunocapture chip. Angew. Chem. Int. Ed..

[40] Bialek J., Muthe A., Yankulov S., Kawan F., Gakis G., Theil G. (2024). Optimizing CTC isolation techniques for molecular characterization of circulating tumor cells in clear cell renal cell carcinoma: A comparative study of EpCAM-based and density-based methods. Cancer Res..

[41] Chowdhury T., Cressiot B., Parisi C., Smolyakov G., Thiébot B., Trichet L., Fernandes F.M., Pelta J., Manivet P. (2023). Circulating tumor cells in cancer diagnostics and prognostics by single-molecule and single-cell characterization. ACS Sens..

[42] Gerratana L., Davis A.A., Foffano L., Reduzzi C., Rossi T., Medford A., Clifton K., Shah A.N., Bucheit L., Velimirovic M. (2025). Integrating machine learning-predicted circulating tumor cells (CTCs) and circulating tumor DNA (ctDNA) in metastatic breast cancer: A proof of principle study on endocrine resistance profiling. Cancer Lett..

[43] Harb W., Fan A., Tran T., Danila D.C., Keys D., Schwartz M., Ionescu-Zanetti C. (2013). Mutational analysis of circulating tumor cells using a novel microfluidic collection device and qPCR assay. Transl. Oncol..

[44] Peeters D., De Laere B., Van den Eynden G., Van Laere S., Rothé F., Ignatiadis M., Sieuwerts A.M., Lambrechts D., Rutten A., Van Dam P. (2013). Semiautomated isolation and molecular characterisation of single or highly purified tumour cells from CellSearch enriched blood samples using dielectrophoretic cell sorting. Br. J. Cancer.

[45] Nagrath S., Sequist L.V., Maheswaran S., Bell D.W., Irimia D., Ulkus L., Smith M.R., Kwak E.L., Digumarthy S., Muzikansky A. (2007). Isolation of rare circulating tumour cells in cancer patients by microchip technology. Nature.

[46] Kang H., Xiong Y., Ma L., Yang T., Xu X. (2022). Recent advances in micro-/nanostructure array integrated microfluidic devices for efficient separation of circulating tumor cells. RSC Adv..

[47] Zhao K., Zhao P., Dong J., Wei Y., Chen B., Wang Y., Pan X., Wang J. (2022). Implementation of an integrated dielectrophoretic and magnetophoretic microfluidic chip for CTC isolation. Biosensors.

[48] Li Q., Wang Y., Gao W., Qian G., Chen X., Liu Y., Yu S. (2024). A microfluidic device for enhanced capture and high activity release of heterogeneous CTCs from whole blood. Talanta.

[49] Wang Y., Wang S., Li L., Zou Y., Liu B., Fang X. (2023). Microfluidics-based molecular profiling of tumor-derived exosomes for liquid biopsy. View.

[50] Macaraniag C., Luan Q., Zhou J., Papautsky I. (2022). Microfluidic techniques for isolation, formation, and characterization of circulating tumor cells and clusters. APL Bioeng..

[51] Addanki S., Meas S., Sarli V.N., Singh B., Lucci A. (2022). Applications of circulating tumor cells and circulating tumor DNA in precision oncology for breast cancers. Int. J. Mol. Sci..

[52] Lee J.H., Park S.H., Kang J., Lee J., Kim S., Kim J., Sohn Y.W., Lee J.K. (2023). Identification of circulating tumor cells based on machine learning. Cancer Res..

[53] Calvo-Almeida S., Serrano-Llabrés I., Cal-González V.M., Piairo P., Pires L.R., Diéguez L., González-Castro L. (2024). Multichannel fluorescence microscopy images CTC detection: A deep learning approach. Proceedings of the International Conference Of Computational Methods In Sciences And Engineering ICCMSE.

[54] Biasiolli L., Ansaloni P., Gentili N., Giardiello D., Montanari F., Miserendino R., Signorini G., Medoro G. (2024). Automated identification and enumeration of CELLSEARCH Circulating Tumor Cells (CTC) with a deep learning algorithm. Cancer Res..

[55] Guo X., Lin F., Yi C., Song J., Sun D., Lin L., Zhong Z., Wu Z., Wang X., Zhang Y. (2022). Deep transfer learning enables lesion tracing of circulating tumor cells. Nat. Commun..

[56] Husseini-Wüsthoff H., Riethdorf S., Schneeweiss A., Trumpp A., Pantel K., Wikman H., Nielsen M., Werner R. (2024). Cluster-based human-in-the-loop strategy for improving machine learning-based circulating tumor cell detection in liquid biopsy. arXiv.

[57] An L., Hu H., Song M., Cheng L., Ba S., Liu Z., Yu Z., Zhang Z., Liu Y., Zhou C.-C. Unsupervised Classification for Circulating Tumor Cells. SSRN 5090990. https://papers.ssrn.com/sol3/papers.cfm?abstract_id=5090990.

[58] Al-Eidi S., Darwish O., Husari G., Chen Y., Elkhodr M. Convolutional neural network structure to detect and localize ctc using image processing. Proceedings of the 2022 IEEE International IOT, Electronics and Mechatronics Conference (IEMTRONICS).

[59] Park J., Ha S., Kim J., Song J.-W., Hyun K.-A., Kamiya T., Jung H.-I. (2024). Classification of circulating tumor cell clusters by morphological characteristics using convolutional neural network-support vector machine. Sens. Actuators B Chem..

[60] Albaradei S., Alganmi N., Albaradie A., Alharbi E., Motwalli O., Thafar M.A., Gojobori T., Essack M., Gao X. (2023). A deep learning model predicts the presence of diverse cancer types using circulating tumor cells. Sci. Rep..

[61] Nanou A., Stoecklein N.H., Doerr D., Driemel C., Terstappen L.W., Coumans F.A. (2024). Training an automated circulating tumor cell classifier when the true classification is uncertain. Proc. Natl. Acad. Sci. USA.

[62] Pastuszak K., Sieczczyński M., Dzięgielewska M., Wolniak R., Drewnowska A., Korpal M., Zembrzuska L., Supernat A., Żaczek A.J. (2024). Detection of circulating tumor cells by means of machine learning using Smart-Seq2 sequencing. Sci. Rep..

[63] Gaysar S., Mustafa Z., Zein A. (2025). Deep Learning Algorithms for Studying the Impact of Tumor Suppressor Gene Mutations on Breast Cancer. J. Clin. Eng..

[64] Wu X., Wang H.-Y., Shi P., Sun R., Wang X., Luo Z., Zeng F., Lebowitz M.S., Lin W.-Y., Lu J.-J. (2022). Long short-term memory model–a deep learning approach for medical data with irregularity in cancer predication with tumor markers. Comput. Biol. Med..

[65] Zhang W., Xu F., Yao J., Mao C., Zhu M., Qian M., Hu J., Zhong H., Zhou J., Shi X. (2023). Single-cell metabolic fingerprints discover a cluster of circulating tumor cells with distinct metastatic potential. Nat. Commun..

[66] Ogut M.G., Ma P., Gupta R., Hoerner C.R., Fan A.C., El-Kaffas A.N., Durmus N.G. (2023). Automated Image Analysis for Characterization of Circulating Tumor Cells and Clusters Sorted by Magnetic Levitation. Adv. Biol..

[67] Srikanth S., Dubey S.K., Javed A., Goel S. (2021). Droplet based microfluidics integrated with machine learning. Sens. Actuators A Phys..

[68] Gangadhar A., Sari-Sarraf H., Vanapalli S.A. (2023). Deep learning assisted holography microscopy for in-flow enumeration of tumor cells in blood. RSC Adv..

[69] Moallem G., Pore A.A., Gangadhar A., Sari-Sarraf H., Vanapalli S.A. (2022). Detection of live breast cancer cells in bright-field microscopy images containing white blood cells by image analysis and deep learning. J. Biomed. Opt..

[70] Ba W., Wang S., Shang M., Zhang Z., Wu H., Yu C., Xing R., Wang W., Wang L., Liu C. (2022). Assessment of deep learning assistance for the pathological diagnosis of gastric cancer. Mod. Pathol..

[71] Surappa S., Multani P., Parlatan U., Sinawang P.D., Kaifi J., Akin D., Demirci U. (2023). Integrated “lab-on-a-chip” microfluidic systems for isolation, enrichment, and analysis of cancer biomarkers. Lab Chip.

[72] Da Col G., Del Ben F., Bulfoni M., Turetta M., Gerratana L., Bertozzi S., Beltrami A.P., Cesselli D. (2022). Image analysis of circulating tumor cells and leukocytes predicts survival and metastatic pattern in breast cancer patients. Front. Oncol..

[73] Liu C., Yang H., Feng Y., Liu C., Rui F., Cao Y., Hu X., Xu J., Fan J., Zhu Q. (2022). A K-nearest neighbor model to predict early recurrence of hepatocellular carcinoma after resection. J. Clin. Transl. Hepatol..

[74] Zhou J., Zhu X., Wu S., Guo J., Zhang K., Xu C., Chen H., Jin Y., Sun Y., Zheng S. (2020). Epithelial-mesenchymal transition status of circulating tumor cells in breast cancer and its clinical relevance. Cancer Biol. Med..

[75] Wei H., Natori T., Tanaka T., Aoki S., Yamada T., Aikawa N. Detection of Circulating Tumor Cells in Blood Using Random Forest. Proceedings of the 2024 International Conference on Electronics, Information, and Communication (ICEIC).

[76] Svensson C.-M., Hübler R., Figge M.T. (2015). Automated classification of circulating tumor cells and the impact of interobsever variability on classifier training and performance. J. Immunol. Res..

[77] Mao Y., Yin Z., Schober J.M. Iteratively training classifiers for circulating tumor cell detection. Proceedings of the 2015 IEEE 12th International Symposium on Biomedical Imaging (ISBI).

[78] Mocan I., Itu R., Ciurte A., Danescu R., Buiga R. Automatic Detection of Tumor Cells in Microscopic Images of Unstained Blood using Convolutional Neural Networks. Proceedings of the 2018 IEEE 14th International Conference on Intelligent Computer Communication and Processing (ICCP).

[79] Ciurte A., Selicean C., Soritau O., Buiga R. (2018). Automatic detection of circulating tumor cells in darkfield microscopic images of unstained blood using boosting techniques. PLoS ONE.

[80] Nissim N., Dudaie M., Barnea I., Shaked N.T. (2021). Real-time stain-free classification of cancer cells and blood cells using interferometric phase microscopy and machine learning. Cytom. Part A.

[81] Zeune L.L., Boink Y.E., van Dalum G., Nanou A., de Wit S., Andree K.C., Swennenhuis J.F., van Gils S.A., Terstappen L.W., Brune C. (2020). Deep learning of circulating tumour cells. Nat. Mach. Intell..

[82] Li X., Chen M., Xu J., Wu D., Ye M., Wang C., Liu W. (2023). Interpretatively automated identification of circulating tumor cells from human peripheral blood with high performance. Front. Bioeng. Biotechnol..

[83] Boya M., Ozkaya-Ahmadov T., Swain B.E., Chu C.-H., Asmare N., Civelekoglu O., Liu R., Lee D., Tobia S., Biliya S. (2022). High throughput, label-free isolation of circulating tumor cell clusters in meshed microwells. Nat. Commun..

[84] Yalamanchili S., Yenuga P., Burla N., Jonnadula H., Bolem S.C. (2024). MRI Brain Tumor Analysis on Improved VGG-16 and Efficient NetB7 Models. J. Image Graph..

[85] Reis H.C., Turk V. (2025). Advanced brain tumor analysis: A novel strategy for segmentation and classification using modern computational methods. Neural Comput. Appl..

[86] Caro-Vegas C., Ramirez C., Landis J., Adimora A.A., Strickler H., French A.L., Ofotokun I., Fischl M., Seaberg E.C., Wang C.-c.J. (2022). Molecular profiling of breast and lung cancer in women with HIV reveals high tumor mutational burden. Aids.

[87] Philip P.A., Azar I., Xiu J., Hall M.J., Hendifar A.E., Lou E., Hwang J.J., Gong J., Feldman R., Ellis M. (2022). Molecular characterization of KRAS wild-type tumors in patients with pancreatic adenocarcinoma. Clin. Cancer Res..

[88] Shahzad M., Ali F., Shirazi S.H., Rasheed A., Ahmad A., Shah B., Kwak D. (2024). Blood cell image segmentation and classification: A systematic review. PeerJ Comput. Sci..

[89] Akbarnataj K., Maleki S., Rezaeian M., Haki M., Shamloo A. (2023). Novel size-based design of spiral microfluidic devices with elliptic configurations and trapezoidal cross-section for ultra-fast isolation of circulating tumor cells. Talanta.

[90] Wang J., Meng X., Yu M., Li X., Chen Z., Wang R., Fang J. (2023). A novel microfluidic system for enrichment of functional circulating tumor cells in cancer patient blood samples by combining cell size and invasiveness. Biosens. Bioelectron..

[91] Shen C., Rawal S., Brown R., Zhou H., Agarwal A., Watson M.A., Cote R.J., Yang C. (2023). Automatic detection of circulating tumor cells and cancer associated fibroblasts using deep learning. Sci. Rep..

[92] Bakhshi M.S., Rizwan M., Khan G.J., Duan H., Zhai K. (2022). Design of a novel integrated microfluidic chip for continuous separation of circulating tumor cells from peripheral blood cells. Sci. Rep..

[93] Edd J.F., Mishra A., Smith K.C., Kapur R., Maheswaran S., Haber D.A., Toner M. (2022). Isolation of circulating tumor cells. Iscience.

[94] Kumar S., Wang Y., Zhan H., Gardner K., Thompson T., Li W. (2024). Classification of Circulating Tumor Cells Using Machine Learning on Microfluidic Trajectory Data. J. ACM.

[95] Zhou M., Zheng H., Wang Z., Li R., Liu X., Zhang W., Wang Z., Li H., Wei Z., Hu Z. (2017). Precisely enumerating circulating tumor cells utilizing a multi-functional microfluidic chip and unique image interpretation algorithm. Theranostics.

[96] Pirone D., Montella A., Sirico D.G., Mugnano M., Villone M.M., Bianco V., Miccio L., Porcelli A.M., Kurelac I., Capasso M. (2023). Label-free liquid biopsy through the identification of tumor cells by machine learning-powered tomographic phase imaging flow cytometry. Sci. Rep..

[97] Gangadhar A., Sari-Sarraf H., Vanapalli S.A. (2022). Staining-free, in-flow enumeration of tumor cells in blood using digital holographic microscopy and deep learning. bioRxiv.

[98] Liang N., Li B., Jia Z., Wang C., Wu P., Zheng T., Wang Y., Qiu F., Wu Y., Su J. (2021). Ultrasensitive detection of circulating tumour DNA via deep methylation sequencing aided by machine learning. Nat. Biomed. Eng..

[99] Muthamilselvan S., Ramasami Sundhar Baabu P., Palaniappan A. (2023). Microfluidics for profiling miRNA biomarker panels in AI-assisted cancer diagnosis and prognosis. Technol. Cancer Res. Treat..

[100] Kim M.W., Kim J.Y., Kim Y., Lee S., Moon S., Hyon J.-y., Hyun K.-A., Yang Y., Ha S., Park S. (2024). Integrating machine learning with microfluidic technologies for proteomic profiling of extracellular vesicles in triple-negative breast cancer. Cancer Res..

[101] Wang C., Wang C., Wu Y., Gao J., Han Y., Chu Y., Qiang L., Qiu J., Gao Y., Wang Y. (2022). High-Throughput, Living Single-Cell, Multiple Secreted Biomarker Profiling Using Microfluidic Chip and Machine Learning for Tumor Cell Classification. Adv. Healthc. Mater..

[102] Noor J., Chaudhry A., Batool S. (2023). Microfluidic technology, artificial intelligence, and biosensors as advanced technologies in cancer screening: A review article. Cureus.

[103] Rejuan R., Aulisa E., Li W., Thompson T., Kumar S., Canic S., Wang Y. (2024). Validation of a Microfluidic Device Prototype for Cancer Detection and Identification: Circulating Tumor Cells Classification Based on Cell Trajectory Analysis Leveraging Cell-Based Modeling and Machine Learning. bioRxiv.

[104] Wang L., Hong R., Shi S., Wang S., Chen Y., Han C., Li M., Ye F. (2024). The prognostic significance of circulating tumor cell enumeration and HER2 expression by a novel automated microfluidic system in metastatic breast cancer. BMC Cancer.

[105] Vora N., Shekar P., Hanulia T., Esmail M., Patra A., Georgakoudi I. (2024). Deep learning-enabled detection of rare circulating tumor cell clusters in whole blood using label-free, flow cytometry. Lab Chip.

[106] Zhu Z., Zhang Y., Zhang W., Tang D., Zhang S., Wang L., Zou X., Ni Z., Zhang S., Lv Y. (2024). High-throughput enrichment of portal venous circulating tumor cells for highly sensitive diagnosis of CA19-9-negative pancreatic cancer patients using inertial microfluidics. Biosens. Bioelectron..

[107] Hua H., Zhou Y., Li W., Zhang J., Deng Y., Khoo B.L. (2024). Microfluidics-based patient-derived disease detection tool for deep learning-assisted precision medicine. Biomicrofluidics.

[108] Pirone D., Montella A., Cavina B., Giugliano G., Schiavo M., Mugnano M., Cerbone V., Scalia G., Porcelli A.M., Gasparre G. (2024). Towards label-free liquid biopsy: Combining machine learning and tomographic phase imaging flow cytometry for the identification of tumor cells. Biomedical Spectroscopy, Microscopy, and Imaging III.

[109] Wu W., Zhang Y., Tan X., Chen Y., Cao Y., Sahai V., Peterson N., Goo L., Fry S., Kathawate V. (2025). Antigen-independent single-cell circulating tumor cell detection using deep-learning-assisted biolasers. Biosens. Bioelectron..

[110] Kouhkord A., Naserifar N. (2025). Ultrasound-assisted microfluidic cell separation: A study on microparticles for enhanced cancer diagnosis. Phys. Fluids.

[111] Liu Z., Zhou Y., Lu J., Gong T., Ibáñez E., Cifuentes A., Lu W. (2024). Microfluidic biosensors for biomarker detection in body fluids: A key approach for early cancer diagnosis. Biomark. Res..

[112] Qi X., Lin S., Li M. (2025). Atomic force microscopy combined with microfluidics for label-free sorting and automated nanomechanics of circulating tumor cells in liquid biopsy. Nanoscale.

